# Unusual Salt and pH Induced Changes in Polyethylenimine Solutions

**DOI:** 10.1371/journal.pone.0158147

**Published:** 2016-09-29

**Authors:** Kimberly A. Curtis, Danielle Miller, Paul Millard, Saswati Basu, Ferenc Horkay, Preethi L Chandran

**Affiliations:** 1 Department of Chemical Engineering, Howard University, Washington, DC, United States of America; 2 Section on Quantitative Imaging and Tissue Sciences, Eunice Kennedy Shriver National Institute of Child Health and Human Development, National Institutes of Health, Bethesda, Maryland, 20892, United States of America; Advanced Centre for Treatment Research and Education in Cancer, INDIA

## Abstract

Linear PEI is a cationic polymer commonly used for complexing DNA into nanoparticles for cell-transfection and gene-therapy applications. The polymer has closely-spaced amines with weak-base protonation capacity, and a hydrophobic backbone that is kept unaggregated by intra-chain repulsion. As a result, in solution PEI exhibits multiple buffering mechanisms, and polyelectrolyte states that shift between aggregated and free forms. We studied the interplay between the aggregation and protonation behavior of 2.5 kDa linear PEI by pH probing, vapor pressure osmometry, dynamic light scattering, and ninhydrin assay. Our results indicate that:

1. At neutral pH, the PEI chains are associated and the addition of NaCl initially reduces and then increases the extent of association.
2. The aggregate form is uncollapsed and co-exists with the free chains.
3. PEI buffering occurs due to continuous or discontinuous charging between stalled states.
4. Ninhydrin assay tracks the number of unprotonated amines in PEI.
5. The size of PEI-DNA complexes is not significantly affected by the free vs. aggregated state of the PEI polymer.

Despite its simple chemical structure, linear PEI displays intricate solution dynamics, which can be harnessed for environment-sensitive biomaterials and for overcoming current challenges with DNA delivery.

## Introduction

Linear polyethylenimine (PEI) is a widely used polymer for packing negatively charged DNA into nanometer-sized particles for cell delivery[1–3]. The polymer consists of amines separated by ethylene groups [4,5]:

$$CH_{2}\text{-}{\left(NH_{2}{}^{+}\text{-}CH_{2}\text{-}CH_{2}\right)}_{n}\text{-}NH_{2}{}^{+}\text{-}CH_{2}\text{-}CH_{3}$$

**Fig 1:**
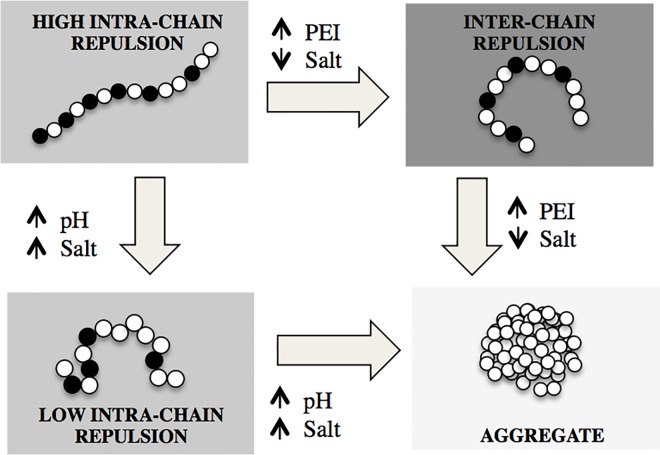
Interactions in a hydrophobic polyelectrolyte that is a weak base. The polymer charge increases with decreasing pH, whereas the charge repulsion is screened by increasing the salt concentration. The backbone extension depends on the balance between inter-chain and intra-chain charge repulsion. Aggregation occurs as intra-chain repulsion is lowered or as inter-chain repulsion is increased. In the figure, the polyelectrolyte chain is depicted as a string of beads, with charged monomers/bead shown in black.

In an acidic environment the PEI chain is positively charged; the charge comes from the protonation of the secondary amines along the backbone. Since the polymer can to take up H^+^ ions, it exhibits weak-base buffering properties that are critical for its application as a DNA-delivery agent. It protects the DNA from the acidic environment of cell-uptake vesicles and ensures the DNA release into the cytoplasm[1,6,7]. PEI is a hydrophobic polymer because of its ethylene-rich backbone. When there is insufficient backbone charge to keep the molecule extended by intra-chain charge repulsion, the polymer collapses or aggregates[8,9]. The conformation of a charged hydrophobic polymer strongly depends on the solution conditions (see Fig 1 and Sec 1A below) [10]. Linear PEI is the simplest hydrophobic, weak-base buffering polyelectrolyte. It is not known how the protonation of PEI is coupled with its hydrophobic-polyelectrolyte characteristics [11].

The backbone extension of a hydrophobic polyelectrolyte depends on the competition between inter- and intra-chain interactions (Fig 1). Intra-chain charge repulsion (i.e., between the amine groups on the same chain) favors chain extension, whereas inter-chain repulsion may compact the molecule. Solution conditions affect the balance between inter- and intra-chain repulsion in the following way: pH increases the charge of the polymer, while added salt reduces charge repulsion due to screening the electrostatic interaction [12]. Increasing PEI concentration increases inter-chain effects. At low-ionic strength the attractive hydrophobic interactions between the polymer segments are often counterbalanced by the electrostatic repulsion, so that an extended molecular conformation is observed. Addition of salt screens the electrostatic repulsion, and the behavior of the solution resembles that of neutral polymers. When the intra-chain repulsion is weak, hydrophobic polyelectrolyte chains collapse. Hydrophobic polyelectrolytes are also suspected to aggregate when the inter-chain repulsion is strong [13]. Charge interactions in polyelectrolyte solution are long-range, and inter-chain charge repulsion may occur at relatively low concentrations. Fig 1 summarizes the states of a hydrophobic polyelectrolyte in different solution conditions.

Unlike in many polyelectrolytes, the charged groups of PEI are located directly on the backbone and separated by only two ethylene groups[14]. Such close spacing of charges has two important consequences. First, the protonation of one amine group will affect the *protonability* of the neighboring amine by increasing the charge-repulsion in its vicinity and therefore the free energy of protonation[11,15]. Secondly, the neighborhood charge repulsion will be sensitive to the extension/aggregation state of the polymer backbone, i.e. its hydrophobic polyelectrolyte properties. The former effect has been observed previously and discussed extensively. For instance, single-chain simulation studies of the intra-chain repulsion forces in PEI indicate that its amine protonation occurs in steps [16]. Smits et al. [14] showed that the titration analysis of PEI required accounting for two- and three- neighbor influence on amine protonation. In fact, it requires 100X more free energy for 50% protonation of amines in the PEI backbone than in its non-polymeric counterpart, dimethyl-amine (apparent pKa of PEI = ~7, dimethylamine pKa ~ 10) [17,18]. However the experimental[19] and computational [16] studies on PEI buffering do not give a detailed profile of how PEI charge changes with pH, and of how its hydrophobic polyelectrolyte state (i.e., presence of aggregation, inter- vs, intra- chain repulsion) alters or is altered by the charging profile.

Understanding how the polyelectrolyte state of PEI influences its protonation/buffering state is important both from a basic polymer-science point of view and for many biological applications of PEI. Linear PEI is a good model for a hydrophobic weak-base polyelectrolyte, because of its simple backbone structure and the absence of side-chains. In the field of polyelectrolytes, the effect of salt concentration and pH have been the subject of many studies, and a quantitative understanding of the role of monovalent ions has developed. However the effect of the hydrophobic backbone on the solution thermodynamics remains much less clear. It is not clear if the aggregated species co-exist with the free polymer and if can they be separated out[12]? Are the aggregates collapsed forms of the polymer, or are they self-assembling clusters? Are they charged species and involved in buffering? And does the buffering capacity of a free polymer change when the charge-repulsion state changes from intra-chain to inter-chain repulsion?

Understanding the coupling between PEI’s protonation and polyelectrolyte behavior is critical for its application as a drug- and DNA-delivery agent. For instance, PEI is functionalized in a number of its drug-delivery applications [20,21]; the degree of functionalization will depend on PEI’s protonated state and the extent of aggregation. Also, it is known that PEI’s charging properties determine its binding with DNA and the stability of the complex in the acidic environment of cell uptake vesicles [4]. However, it is not clear if the charging properties are altered by the polyelectrolyte state of the polymer. Moreover, the free and aggregate forms of PEI have different biological toxicities [22–24]. The former disrupts cells and cellular vesicles by inserting into their negative-charged lipid membranes. The latter sequesters opposite-charged proteins and entities in the blood stream and renders them ineffective for delivery. Strategies for reducing the toxicity of PEI would need to account for its polyelectrolyte state in different solution conditions. Finally, given the increasing interest in PEI-based biomaterials, the polyelectrolyte state of PEI can be exploited for novel, application-specific biomaterial designs. Despite the widespread biological use of PEI no systematic experimental studies have been made on its polyelectrolyte behavior at polymer and ion concentrations and at pH that are relevant in the biological milieu.

The goal of this work is to establish the hydrophobic-polyelectrolyte behavior of PEI and its effect on the polymer’s protonation profile. We study the effects of PEI concentration, NaCl concentration, and pH on the association of PEI chains by Vapor Pressure Osmometry and Dynamic Light Scattering (DLS). The latter technique allows us to determine the hydrodynamic radius of the polymer and to separate the contributions of the two PEI forms (free chains and associations). Specific attention was given to NaCl because of its relevance in biological systems. The protonation state is tracked from the polymer’s uptake of H^+^ or OH^-^ ions. Our pH titration experiments are different from those published in the literature. In our study both salt and PEI concentrations are maintained constant for all H^+^ additions. A low molecular weight linear PEI (2.5 kDa) was chosen to minimize variations due to polydispersity, Also, longer and flexible hydrophobic polyelectrolytes can form collapsed domains along the backbone, which makes the relation between the hydrodynamic radius and backbone extension difficult to interpret [8,13]. Atomistic simulations of shorter 2.5 kDa linear PEI does not show collapsed backbone domains. [25]. Therefore, changes in the hydrodynamic radius of the free PEI polymer can be interpreted as changes in the root-mean-square radius of the polymer or as its backbone extension/compaction.

## Materials and Methods

### Sample Preparation

#### Preparation of PEI solutions

PEI (2.5kDa, Polysciences Inc) was mixed with water (1 μm sterile-filtered and molecular biology grade, Sigma Aldrich, St. Louis, MO) to obtain a final PEI concentration of 13.6 mM in amine groups. The mixture was dissolved by heating to ~80°C and adding HCl to reduce the pH to ~7.5. The 13.6 mM stock solution was sterile-filtered (Acrosidic 32 mm Syringe Filters with 0.2 μm Supor membrane, Pall Corporation, MD) for subsequent use.

Along with every PEI stock solution, a control polymer-free solution was prepared that was subjected to the same HCl additions and heat treatments as the stock. Every subsequent dilution, salt addition, and pH modification that was performed on the PEI stock, was also performed on aliquots of the polymer-free solution. These polymer-free solutions were used as the controls for the osmotic and light scattering experiments performed on the corresponding PEI solutions.

#### Preparation of DNA nanoparticles

PEI solutions of different salt content and pH were prepared and equilibrated overnight. Salt-free DNA in water (kind gift from Dr. Anna K. Allen) was added to the PEI solutions to achieve final concentration of 2ng/l DNA. Nanoparticles were formed as the DNA packed in the PEI. The solution was incubated at room temperature overnight and the nanoparticles’ hydrodynamic radii were measured by Malvern Zetasizer ZS.

#### Sample Preparation for Ninhydrin assay

100 mM aqueous solution of ninhydrin reagent (Sigma Aldrich, New York) was added to 3 ml of PEI solution (concentration: 1–8 mM) to obtain a final ninhydrin concentration of 3 mM. The solution was vortexed vigorously for 1 minute, and kept in a hot water bath (70–80°C) for 20–25 minutes. A yellow-orange color develops due to the reaction between ninhydrin and secondary amines. The solution tubes were then placed in a cold water bath (5°C) for ~10 minutes and the absorbance at 487 nm was measured with a UV-Visible spectrophotometer (Cary 5000 UV-Vis NIR spectrophotometer, Varian Inc, CA). The color was stable for ~24 hours.

### Measurement Techniques

#### Osmotic Pressure Measurements

The osmotic pressure of PEI solutions was measured by a Knauer K-7000 Vapor Pressure Osmometer. The osmometer contains two thermistors: a drop of solution was placed on one of the thermistors and a drop of solvent on the other thermistor. Solvent vapor is condensed into the solution because the vapor pressure of the solvent in the solution is smaller than in the pure solvent. The condensation released heat and resulted in a temperature difference between the two thermistors. This temperature difference was detected by measuring the microvolts imbalance on a Wheatstone bridge circuit. In solutions of non-associating solutes the temperature difference is proportional to the number of dissolved particles and is given by c/M (see Eq 1). In associating solutions, however, Eq 1 breaks down, and the osmotic pressure exhibits either a plateau or a maximum as a function of the polymer concentration.

Vapor pressure osmometry is a rapid and precise method that provides information about the state of aggregation and also about interactions in solutions of relatively low molecular weight polymers (M < 10 kDa). The osmotic properties of dilute polyelectrolytes in the presence of added salt are determined by two parameters: the molecular weight of the polymer (M), and the second virial coefficient (B_2_), which is governed by the interaction between the polymer molecules of the solute and the solvent. In dilute solutions, in which the polyelectrolyte chains are molecularly distributed, the osmotic pressure П is given by Eq 1

$$\Pi =\left(\frac{RT}{V_{1}}\right)\left(\frac{c}{M}+B_{2}c^{2}+B_{3}c^{3}+..\right)$$ (1)

where c is the polymer concentration, B_3_ is the third virial coefficient, R is the gas constant, T is the absolute temperature and V_1_ is the partial molar volume of the solvent.

The osmotic pressure measurements were made at 25°C.

#### Dynamic Light Scattering (DLS)

DLS measurements of 1 ml PEI solutions in quartz cuvettes (Malvern Instruments, Inc., Westborough, MA) were performed in a Zetasizer ZSP (Malvern Instruments, Inc., Westborough, MA) at 633 nm wavelength and 173° scattering angle. Measurements with multiple scattering angles were performed with a Precision Detector—Expert Laser Light Scattering DLS Workstation equipped with a HeNe laser (wavelength: 698 nm). All samples were then equilibrated at 25°C for 30 minutes in the light scattering apparatus before measurements. The duration of data collection was 2500 sec because of the relatively low polymer concentration of the PEI solutions. Laser attenuation, sampling position, and sampling time were maintained constant for all measurements.

In DLS, the auto-correlation of the intensity of scattered light, G(t)-1, is recorded as a a function of time (t). The time scale over which the correlation decays is the relaxation time **τ**. When there are multiple relaxation times, the intensity correlation curve shows multiple falls. The correlation function is defined by

$$G(t)-1=\sum_{i}A_{i}\;exp\left(\frac{-t}{\tau_{i}^{\gamma_{i}}}\right)$$ (2)

where A_i_ and γ_i_ are the corresponding intensity contribution, and polydispersity. A_i_ is related to the size and number of scattering species. γ_i_ is related to the spread of the hydrodynamic diameter, and had values between 0.7 and 0.85 in our experiments. A value of 1 indicates no spread whereas a value of 0.6 indicates that the spread is probably due to multiple unresolved peaks. The polydispersity observed in our light scattering experiments is in the range expected for the polydispersity index (i.e., M_w_/M_n_) of 1.2 reported by the manufacturer of the PEI powder.

The DLS correlation curve from a typical PEI solution displays two characteristic relaxation times (one due to the free polymer (f) and the other due to the aggregated form (a) [26]. The DLS parameters corresponding to both forms are obtained from Eq 3

$$G(t)-1=A_{f}\;exp\left(\frac{-t}{\tau_{f}^{\gamma_{f}}}\right)+A_{a}\;exp\left(\frac{-t}{\tau_{a}^{\gamma_{a}}}\right)$$ (3)

The fast relaxation time is related to the diffusion coefficient D by

$$\tau_{i}^{-1}=D_{i}{\left(\frac{4\pi n_{o}}{\lambda }sin(\theta /2)\right)}^{2}$$ (4)

where **λ** is the wavelength of the incident light, n is the refractive index of the solvent, and **θ** is the scattering angle. The hydrodynamic radius (R_h_) can be determined from the Stokes-Einstein equation

$$\tau_{i}^{-1}=k_{B}T{\left(\frac{4\pi n_{o}}{\lambda }sin(\theta /2)\right)}^{2}/6\pi \eta R_{h}^{i}$$ (5)

where k_B_ is the Boltzmann constant and **η** is the solvent viscosity. The experiments were performed at 25°C, and the viscosity of water at 25°C was assumed with correction for the supplemented NaCl.

We report the hydrodynamic radius R_h_ of the free polymer to monitor the extension state of the molecule. The distribution between the free and aggregated states is estimated from the intensity contributions A_f_ and A_a_.

#### Zeta Potential

Zeta Potential of the PEI solutions with only aggregates present were determined with the Malvern Zetasizer ZSP using 1 ml disposable cuvettes, and measurement parameters of 300 sec runtime and 6 runs per sample.

#### pH titration and calculation of protonation fraction

Fixed volumes of HCl/NaOH with logarithmically increasing molarity were added to separate samples of PEI and NaCl solutions so that the final PEI/NaCl concentration was kept constant. The pH measurements were undertaken after at least 2 hours equilibration using a ThermoScientific Orion pH meter fitted with a Ross Micro probe. There are two important differences between our pH titration method and that typically made in polyelectrolyte solutions. In most polyelectrolyte titrations the polymer is first completely charged with the addition of a base/acid and then the completely charged polymers are titrated [14]. We did not follow this method because charging the PEI polymer with HCl would also increase its counter-ion content, and would render the solutions not optimal for the low salt concentration experiments. Also, typical titrations involve adding acid/base of fixed molarity. This would dilute the PEI and salt concentration during the titration. In order to maintain the PEI and NaCl concentrations constant, we performed the titration by adding fixed volumes of HCl/NaOH of logarithmically increasing molarity to *separate* samples of PEI solutions.

***Analysis***: The amount of H^+^ ions taken up by the PEI solution was determined from the difference between the added H^+^ ions and the H^+^ ions remained free in the solution (pH). The four reactions occurring in the PEI solution are

$$\left[NaCl]\to [Na^{+}]+[Cl^{-}\right]$$ (6)

$$\left[HCl]\to [H^{+}]+[Cl^{-}\right]$$ (7)

$$\left[H_{2}O]\Leftrightarrow [H^{+}]+[OH^{-}\right]$$ (8)

$$\left[A]+[H^{+}]\Leftrightarrow [AH^{+}\right]$$ (9)

The disassociation of water (Eq 8) is governed by the equilibrium constant k_w_ = [H^+^][OH^-^] = 10^−14^. The charge balance (electroneutrality) requires that

$$\left[AH^{+}]+[H^{+}]+[Na^{+}]=[OH^{-}]+[Cl^{-}\right]$$ (10)

where [A] and [AH^+^] are the concentrations of the unprotonated and protonated amines. There are two sources for Na^+^ and Cl^-^ ions: first from the NaCl added to the PEI solution, and second from the HCl/NaOH added during titration. HCl was also added initially to the PEI stock solution to dissolve the salt-free PEI powder. Specifying the source of ions in Eq 9, we get

$$[AH^{+}]+{\left[H^{+}\right]}_{free}+{\left[Na^{+}\right]}_{salt}+{\left[Na^{+}\right]}_{NaOH}={\left[OH^{-}\right]}_{free}+{\left[Cl^{-}\right]}_{salt}+{\left[Cl^{-}\right]}_{HCl}$$ (11)

The charge contributions from the added NaCl cancel each other giving

$$[AH^{+}]={\left[OH^{-}\right]}_{free}+{\left[Cl^{-}\right]}_{HCl}-{\left[H^{+}\right]}_{free}-{\left[Na^{+}\right]}_{NaOH}$$ (12)

where the free [H^+^] and [OH^-^] are known from the pH of the solution. The protonated fraction of the PEI solution can then be obtained

$$P=[AH^{+}]/{\left[A\right]}_{0}$$ (13)

where [A]_o_ is the total amine concentration of the solution, and [AH^+^] is given by Eq 12.

In order to correct for the H^+^ ions coming from CO_2_ present in distilled water, the overhead space of the PEI solutions was minimized and filled with Nitrogen. Also, controls without PEI were made for each titration sample to keep track of the H^+^ concentration in the absence of PEI buffering.

## Results and Discussion

### Effect of aggregation on the osmotic pressure of PEI in NaCl solutions

**Fig 2:**
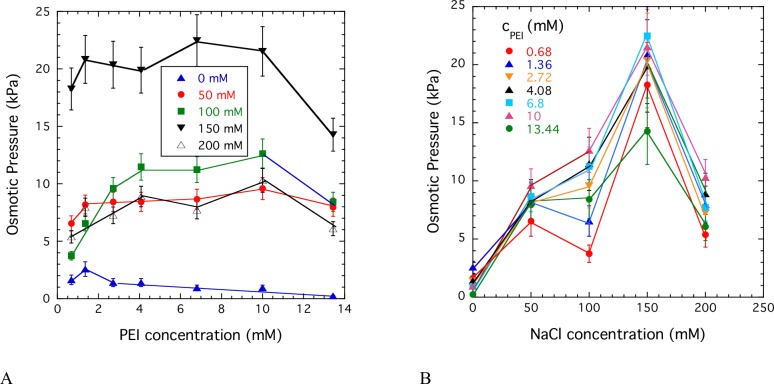
Variation of the osmotic pressure of PEI solutions as a function of the polymer concentration at constant NaCl concentrations (A), and as a function of the NaCl concentration at constant polymer concentrations (B).

Osmotic pressure measurements give information on both the state of aggregation of the polymer molecules and the effect of salt on the thermodynamic interactions. Fig 2A shows the variation of the osmotic pressure as a function of the PEI concentration for solutions with constant NaCl concentrations. The shape of all curves is qualitatively similar. At low PEI concentration the osmotic pressure increases with the polymer concentration and approaches a plateau at around 2 mM < c < 4 mM PEI concentration. The observed behavior is typical of associating solutions in which the polymer molecules aggregate due to polar or ionic interactions or hydrogen bonding. In such solutions free polymer chains coexist with large clusters. The results shown in Fig 2B suggest that small quantity of NaCl prevents PEI aggregation and increases solubility. The solubility of the present 2.5 kDa PEI reaches a maximum at 150 mM NaCl concentration. The decrease of the osmotic pressure at higher salt concentration (c > 150 mM) can be attributed to screening of the electrostatic repulsion by the added salt.

### PEI polyelectrolyte state during dissolution in physiological pH range

#### Salt concentration changes distribution between aggregate and free PEI forms

**Fig 3:**
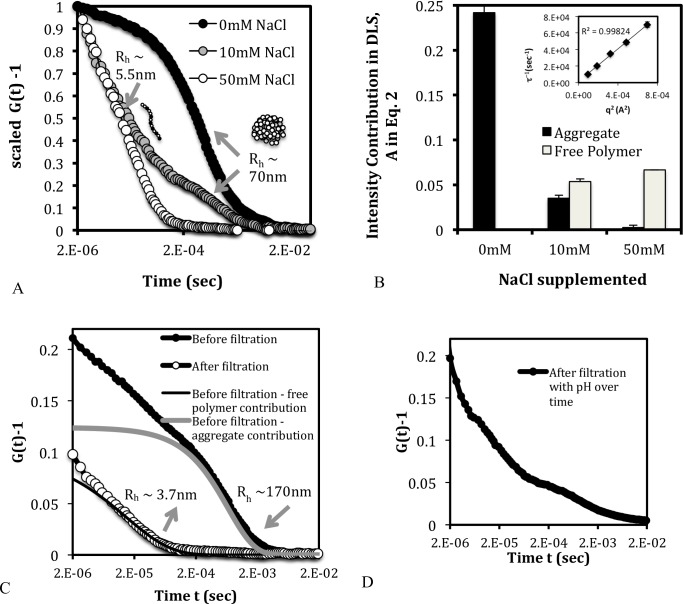
Distribution of the aggregated and free polymer species at PEI dissolution and with addition of NaCl. (A) Scaled DLS correlation data showing the predominance of the aggregate form in the absence of NaCl, and the gradual release of free polymer with the addition of salt. The arrows point to the hydrodynamic diameters corresponding to the decay region of the DLS curve. (B) Unscaled intensity contribution showing that the aggregate form releases free polymers as salt is added to the solution. (C) DLS correlation curve showing that filtration removes the contribution from the aggregate form, indicating that the latter is a separable entity. (D) DLS correlation curve of the solution from C over time with the addition of HCL in this case, showing the return of the aggregate contribution. The data in C and D are shown for 2.72 mM PEI and 50 mM NaCl.

To determine the size of the aggregates we made DLS measurements on a 2.72 mM PEI solution (Fig 3A). In the absence of added NaCl, the solution showed only one relaxation time corresponding to R_h_ ~140 nm. This size is much larger than that expected for a 2.5 kDa PEI polymer (contour length ~19 nm) indicating that the diffusing entities are large aggregates of many PEI chains. When 10 mM NaCl was added, a faster relaxation mode appeared with R_h_ ~ 5nm (Fig 3A), which is within the expected range for free 2.5 kDa polymer. The addition of salt seems to release free polymer from the aggregates (Fig 3A). By 50 mM NaCl concentration only one relaxation time due to the free polymer is observed. The interconversion between aggregate and free polymer molecules (Fig 3B) suggests that the former is not in a collapsed state but coexists with the free polymer. The relaxation rate of the free polymer **τ**^-1^ q was measured at different scattering angles **θ** and plotted against q^2^, where q is the scattering wavelength given by

$$q=\left(\frac{4\pi n_{o}}{\lambda }sin(\theta /2)\right)$$ (14)

The linear dependence between **τ**^-1^ and q^2^ (Fig 3B, inset) confirms that the free polymer is a diffusing species as modeled by Eq 4. To check if the aggregate is a removable species, we filtered a PEI solution containing both free and aggregate forms (Fig 3C). Filtration through a 200 nm pore size filter removed the species with d_H_~ 340 nm and greater. The disappearance of this slow relaxation component indicates that the aggregates are near-completely separable by filtration. Only the fast contribution from the free polymer remained after filtration, *with the relaxation rate and intensity contribution similar to that before filtration* (Fig 3C). Over time, the aggregates reappeared when the pH of the solution was increased (Fig 3D). The latter observation indicates that PEI aggregates are not only uncollapsed and removable entities, but are also in dynamic equilibrium with the free polymer.

#### The DLS data are qualitatively consistent with the osmotic observations

The osmotic pressure data indicates that the number of free (mobile) entities increases with the addition of salt from zero to 50 mM. Correspondingly in the DLS data, the contribution from free polymers increases as salt concentration increases to 50 mM. Together the two experiments imply the release of free polymers from aggregates as the salt concentration increases. The aggregation behavior of PEI is unusual; aggregates can only exist if there is a net attraction between the molecules. However PEI polyions have the same electric charge, and therefore should repel each other instead of forming aggregates. It is likely that in the present solutions attractive interactions of nonelectrostatic origin play a role in the formation of PEI aggregates.

#### Apparent protonation state of aggregate is similar to free polymer form

PEI was purchased in salt-free powder form. In this state the PEI polymer is unprotonated, and therefore hydrophobic and undissolvable in plain water. HCl is typically added to dissolve the polymer. From the difference in the amount of HCl added and the amount of H^+^ remaining in solution (i.e., the pH), it was estimated that the PEI solution needed to be ~33% charged for dissolution to occur. At the physiological pH of ~7.5, the polymer is about 44% charged and dissolved. The salt effect on protonation was studied at pH 7.5. We observed that as NaCl was added to the PEI solution, the pH did not change significantly, even though the distribution between free and aggregated polymers changed (Fig 3). In other words, significant amount of H^+^ ions (on the order of the amine concentration) were neither taken up nor released as aggregates were converted to free chains. Therefore, one can conclude that both the PEI aggregates and free polymer forms of PEI have the same charge ratio at ‘neutral’ pH. Our report of 44% charge ratio for the free polymer is in the range reported in other studies [14,16]. However, this is the first time the charge of the aggregates has been reported and it is ~44% at neutral pH.

### Hydrophobic-polyelectrolyte regimes of PEI in the neutral pH range

#### Added salt produces a biphasic behavior in free-polymer R_h_

**Fig 4:**
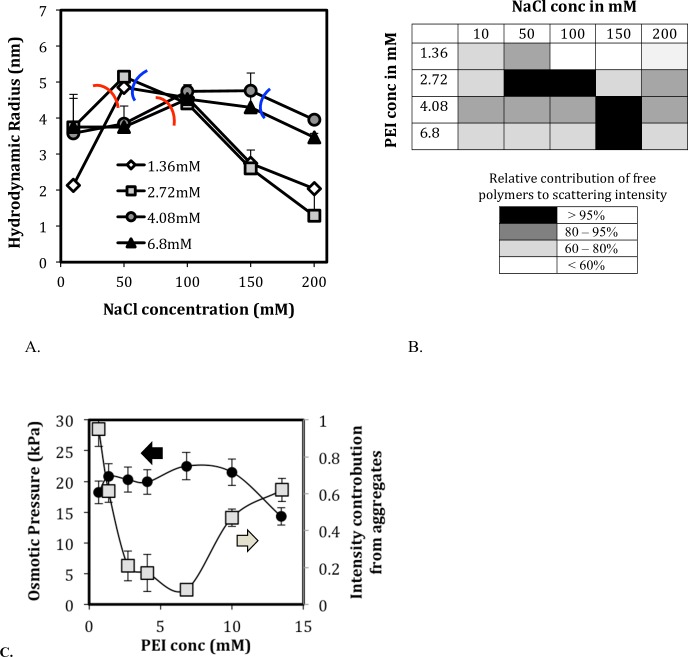
Hydrophobic polyelectrolyte dynamics of PEI in the neutral pH range (pH 7–8). (A) The hydrodynamic radius of PEI shown as a function of NaCl concentration for different PEI concentrations (legend). The hydrodynamic radius increases in the inter-chain repulsion regime and decreases in the intra-chain repulsion regime. (B) Distribution of free and aggregated PEI forms at neutral pH range with varying PEI and NaCl concentration. The free polymer content increases with salt concentration and decreases again. The salt concentration required for maximum free polymer (black) increases with PEI concentration. (C) In DLS, the level of aggregation initially decreases with PEI concentration and then increases. Correspondingly in osmotic experiments, the number of diffusing species increases with PEI concentration then decreases.

Fig 4A shows the hydrodynamic radius, R_h_, of the free polymer as a function of the concentration of the added salt. The R_h_ does not decrease monotonically with salt as is typically observed in polyelectrolyte solutions. Instead, R_h_ initially increases and then decreases. The pH remains within the range of 7–8, indicating that there is less than a 1% change in the apparent PEI protonation for the different salt and polymer concentrations. A possible reason for the initial increase and then decrease of R_h_ can be attributed to the salt-screening effect schematized in Fig 1. The addition of salt initially screens inter-chain repulsions that tend to extend the polymer (left of red curve in Fig 4A) and then proceeds to screen intra-chain repulsions that tend to compact the polymers (right of blue curve). Fig 4B shows the distribution of aggregated and free polymers in solutions of Fig 4A, where the darker colors denote larger amount of free polymers (Fig 4B). For a given PEI concentration, the amount of free polymer increases with the salt content (as was also observed in Sec. 3A) and then decreases again. The trend is consistent with the osmotic data of Fig 2A where the number of diffusing entities (i.e. free polymers) initially increases and then decreases with the addition of salt. This change in the free polymer contribution also nearly tracks the inter- and intra- chain repulsion regimes in Fig 4A. The level of aggregation is minimum (i.e. free polymer contribution > 95%) in solution conditions where intra-chain repulsion is highest (between the blue and red curves in Fig 4A). The osmotic and DLS results both show the biphasic dependence of the aggregation levels on the PEI concentration (Fig 4C). We note that all solutions in a given experiment were prepared from the same stock, and the dynamic redistributions between aggregates and free polymers are consistent with two forms coexisting in equilibrium.

It is not clear what interactions are involved in the reversible formation of PEI aggregates. Polymer aggregation is a complex interplay between electrostatic (coulombic) and nonelectrostatic interactions. Electrostatic interactions are associated with electrolytes, which are ionized. These interactions can be either attractive or repulsive, and they strongly depend on the charge density of the components (e.g., aggregates and dissolved molecules), as well as the ionic strength of the solution. Nonelectrostatic interactions are always attractive; they include van der Waals forces, hydrophobic interactions and hydrogen bonding. In the PEI solution the polymer chains are identically charged and, therefore, repel each other. However, our experimental findings clearly indicate the presence of large aggregates. Therefore it is natural to attribute the aggregation to attractive (nonelectrostatic) interactions, probably mainly arising from hydrophobic interactions of the ethylene (-CH_2_-CH_2_-) groups of the PEI molecule. But also hydrogen bonding might be involved, which commonly occurs when a hydrogen atom is bound to a highly electronegative atom (nitrogen, oxygen, etc.).

### Polyelectrolyte states in protonation regimes

#### Polyelectrolyte state does not affect PEI protonability over a wider pH range

**Fig 5:**
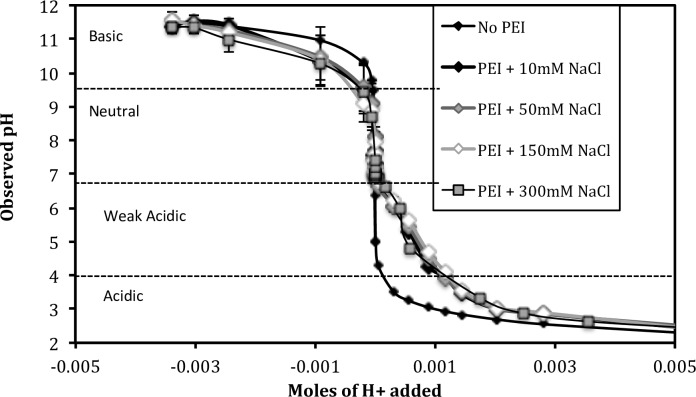
pH titration curves of 2.5kDa PEI at 4.08 mM PEI at different salt concentrations. The polymer and salt concentration was maintained constant for all titration samples. The polyelectrolyte-protonation underlying the titration curve is studied in the context of four pH regions.

Fig 5 shows the pH titration curve of 4.08 mM PEI for a range of salt concentrations (10, 50, 150, 300 mM NaCl). As described in the Methods Section, each H^+^/OH^-^ addition was performed on separate samples in order to maintain both PEI and NaCl concentrations constant. The H^+^ concentration in the x-axis does not include the H^+^ ions added during dissolution of the stock solution. The polyelectrolyte state of 4.08 mM PEI at neutral pH changes as the salt concentration increases from 10 to 300 mM NaCl. The aggregation level varies from ~20% to ~5% and then goes back to ~20% (Fig 4B). However, there is no significant difference in the shape of the titration curves. The relative salt-independence of the titration profile indicates that the protonation or charge ratio of PEI (given by the titration profile) is unaffected by the levels of aggregation and the intra- vs. inter- chain charge repulsion (determined by the salt concentration).

To our knowledge, there is no comparable titration data for linear PEI in the literature. Smits et al.[14] performed titrations on linear PEI with a potentiometer, but both the concentration and molecular weight of their PEI were significantly higher (22 mM and 44 kDa, respectively), and the concentration changed with each acid/base addition. The authors did not observe significant changes in the shape of the titration curve as the salt concentration increased from 0 – 1M in steps of 100 mM. Our results are qualitatively consistent with these findings. The reason for the salt independence of the titration profile is not known, and simulation models predict otherwise [16].

The linear PEI titration curves in Fig 5 show two pKa. The pKa of ~4.5 can be attributed to the protonation of the free polymers which are the abundant species in acidic regime. The pKa of ~10 can be attributed to the protonation of the aggregates which are the abundant species in basic regime (see below). Researchers have reported two pKa for *branched* PEI, also in the acidic and basic regions of the titration curve. However these two pKa have been attributed to the protonation of the tertiary and secondary amines of the branched PEI, respectively.

### Polyelectrolyte-protonation dynamics during pH titration

**Fig 6:**
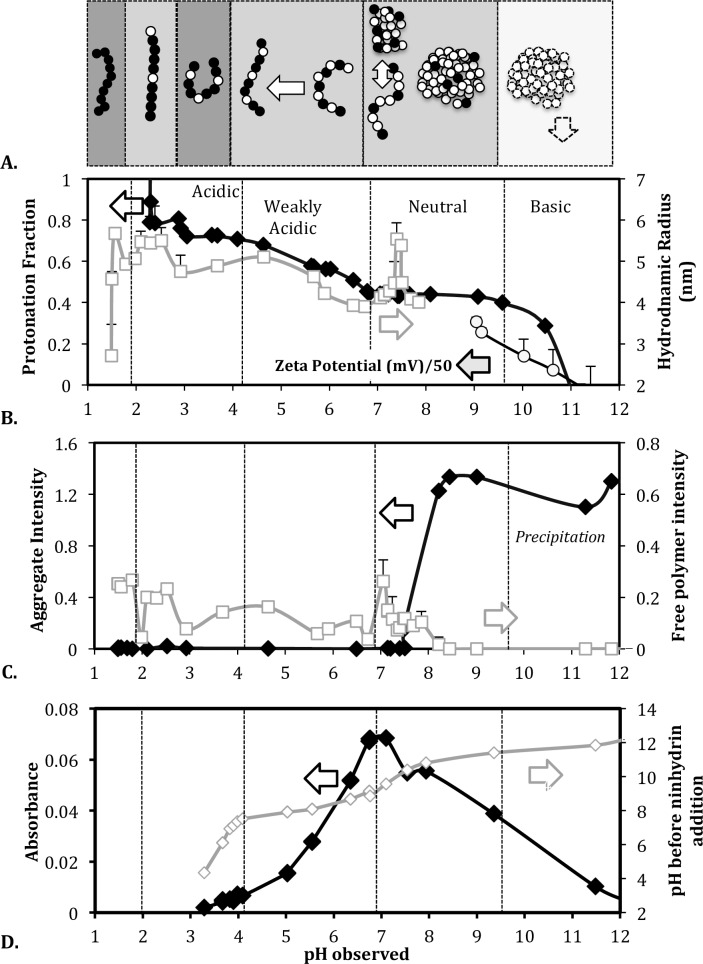
pH dependent protonation-polyelectrolyte interplay in 4.08mM PEI solution with 150mM NaCl. (A) Schematic depicting the protonation and polyelectrolyte state of the polymer in each pH region. The grey shading in each region represents the different charge repulsion regimes described in Fig 1. Charged PEI monomers are shown as black beads and uncharged monomers as white beads. (B) Simultaneous tracking of PEI protonation and backbone extension state in different pH regions. (C) Contribution of the free and aggregated PEI forms to the scattering intensity. (D) Changes in the pH and absorbance of PEI solution at 440 nm following acid and ninhydrin additions.

We investigated how the protonation and polyelectrolyte state changed during the pH titration of the 4.08 mM PEI solution (Fig 6). We focused on the 150 mM NaCl sample where the polymer has high intra-chain repulsion and is present mostly in the free polymer state at neutral pH as indicated by the osmotic pressure and DLS results. The H^+^ uptake was monitored by calculating the protonation ratio for each sample (Eq 11) (Fig 6B). The polyelectrolyte state was tracked by following the intensity contribution from the free and aggregated PEI forms (Fig 6C), and the hydrodynamic diameter of the free polymer (Fig 6B). To enable meaningful comparison of intensity contributions, the DLS laser attenuation and sampling position were maintained constant for all samples. The results are discussed in terms of four pH regions. The grey shading of each pH region in Fig 6A corresponds to the charge-repulsion regime with the same shading as in Fig 1.

Basic region (~12 > pH > 9.5): Beyond pH = 9.5, the aggregate is the dominant form (Fig 6C) and its charge falls from ~44% to negative values (Fig 6B). The decrease in the aggregate’s positive charge is also reflected by the zeta potential which falls from 7± 4 mV at pH = 10 to near 0 mV at pH = 11, where the polymer precipitates.

Neutral region (~9.5 > pH > 6.8): In this region, the net PEI protonation remains constant at 44%. The aggregate is the only form present at pH = 9.5, and it gradually converts to free chains as the pH reduces from 9.5 to ~7 (Fig 6C). The extent of aggregate to free chain conversion depends on the salt concentration (Figs 4 and 5). The zeta potential of the aggregate at pH = 9 is about 15.3 ± 1.1 mV. Interestingly, the zeta potential is in the range typically observed for dispersed DNA-PEI nanoparticles [27,28]. Free polymer chains become detectable below pH = 8 and their hydrodynamic radii exhibit a maximum at around pH 7.5.

Weak Acidic region (~6.8> pH > ~4): In this region, the free PEI chains dominate the scattering response (Fig 6C). Buffering is observed (Fig 5) as the polymer protonation increases steadily from ~44% to ~70% (Fig 6B). The hydrodynamic diameter of the free polymer increases with protonation, which is expected due to the intra-chain repulsion in the increasingly charged polymer.

Acidic region (~4 > pH > 2): In this region the buffering capacity decreases while PEI protonation remains nearly constant at 66–70% (Fig 6B). The hydrodynamic radius decreases, suggesting that the free polymer chains are gradually compacted possibly due to interchain repulsion between the highly charged polyions [14]. Below pH = 3 the protonation rapidly increases and reaches ~95% (Fig 6C). Correspondingly, R_h_ exhibits a peak value and remains constant. Beyond pH = 2, there is no significant buffering since the polymer has reached its maximum protonation (Figs 5 and 6C).

#### PEI buffering by continuous or discontinuous protonation between stalled charge states

**Fig 7:**
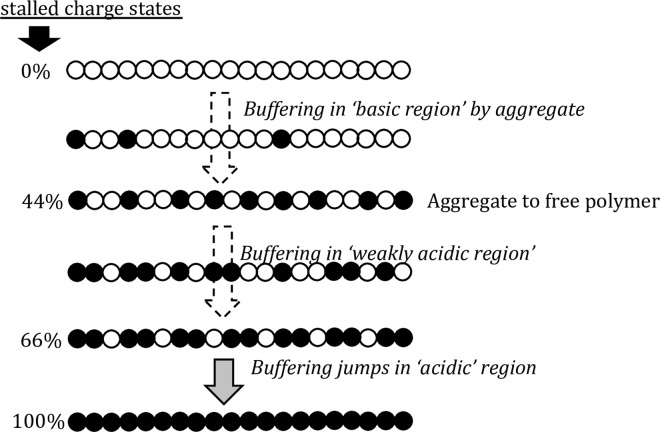
Schematic depiction of the stalled protonation states of PEI between which protonation/buffering occurs continuously and discontinuously.

The main features of the protonation profile are consistent with the observations reported by Smits et al. [14]. The authors found that PEI protonability was not symmetric on either side of the 50% charging point. The asymmetry was attributed to the doublet and triplet amine interactions that occur above 50% charge. We observed similar changes in protonability on either side of the ‘neutral’ pH region. Smits at al. also reported that protonability becomes difficult at about 2/3^rd^ protonation ratio, and is accompanied by a large increase in solution viscosity. The viscosity increase was reversed by charge-screening suggesting that inter-chain interactions occur in this region. We made similar observations in the ‘acidic’ region where the protonation stalls at ~70% charge. The stall could be occurring because further protonation may involve triplet interactions [14] (Fig 7).

### PEI protonation/buffering profile reflected in ninhydrin interaction

The ninhydrin assay is popularly used for quantifying the secondary amines in linear PEI [29]. However the reaction mechanism implies that it quantifies unprotonated secondary amines, and this was used to verify the pH dependence of PEI’s protonation (Fig 6A). The assay was performed on 4.08 mM PEI solutions in 150 mM NaCl at various pH. Secondary amines react with ninhydrin in acidic medium to give iminium salt [30]. The iminium salt has a characteristic yellow color with optimum UV-Vis absorbance at 440 nm.

#### Response to acid addition is consistent with PEI buffering/protonability in each pH region

During the assay a fixed amount of acid is added to all PEI solutions. The pH of the PEI solutions before and after acid addition are shown in Fig 6D (plot with unfilled diamonds). The slope of the plot reflects the buffering capacity of the polymer. For instance, the slope is lower in the ‘weak acidic’ and ‘basic’ regions where the buffering capacity is high, and the slope is high in the ‘neutral’ and ‘acidic’ regions where the buffering capacity is low. In the ‘weak acidic’ and ‘basic’ regions, the free polymer buffers the removal of H^+^ ions by changing its protonation; therefore, the solution pH changes slowly. In the ‘neutral’ and ‘acidic’ regions the polymer protonation state does not vary notably, and therefore the slope is greater.

#### Extent of ninhydrin reaction is consistent with PEI protonation profile

The formation of the iminium salt requires both a transferable electron pair on the amine nitrogen and an acidic medium. The reaction between ninhydrin and a secondary amine to form the iminium salt proceeds in two stages: (1) the lone-pair of electrons from the nitrogen of PEI’s secondary amine is transferred to the ninhydrin complex; and (2) the ninhydrin complex undergoes hydrolysis in the acidic medium to form iminum salt [30]. Acidic pH decreases iminium salt formation in linear PEI (i.e., absorbance decreases in the ‘weak acidic’ region, Fig 6D), which can be attributed to the decrease in the number of nitrogen atoms being able to donate lone-pair of electrons as they become protonated. In other words the absorbance, and therefore the iminium salt formation, should track the PEI protonation profile as demonstrated in Fig 6D. The absorbance changes slowly around pH = 7 where the protonation stalls at 44%, falls rapidly from pH = 7 to pH = 4 (‘weakly acidic’ region) where the polymer protonation increases, and changes slowly from pH = 4 to pH = 3, where the polymer protonation stalls again, and becomes negligible beyond pH = 3 where the polymer protonation is near complete.

#### Effect of the PEI protonation/buffering profile on DNA interaction and nanoparticle packing

Our results indicate that PEI exists in two forms and the size of the free polymer chain depends on the salt concentration and the nature of charge repulsion. The level of protonation of the polymer can be controlled by the pH of the solution. In the context of DNA delivery application it is essential to know how the protonation/polyelectrolyte state of PEI affects its interaction with DNA and the subsequent formation of DNA-PEI nanoparticles [31]. Previous studies have shown that the DNA persistence length, aggregation, and charge (pKa of DNA phosphate groups is ~0) only weakly vary in the range of pH and monovalent-ion concentrations used in this study [32,33][34]. The PEI polymer, however, shows large changes in charge, size, and aggregation within the same salt and pH range. Therefore we tracked the size of the DNA-PEI complexes to check if it correlated with protonation/aggregation state of PEI. Such correlation would indicate an obvious dependence between the PEI state and the DNA-PEI interactions leading to nanoparticle packing.

#### Size of DNA-PEI nanoparticles and therefore DNA-PEI packing is unaffected by PEI aggregation state

**Fig 8:**
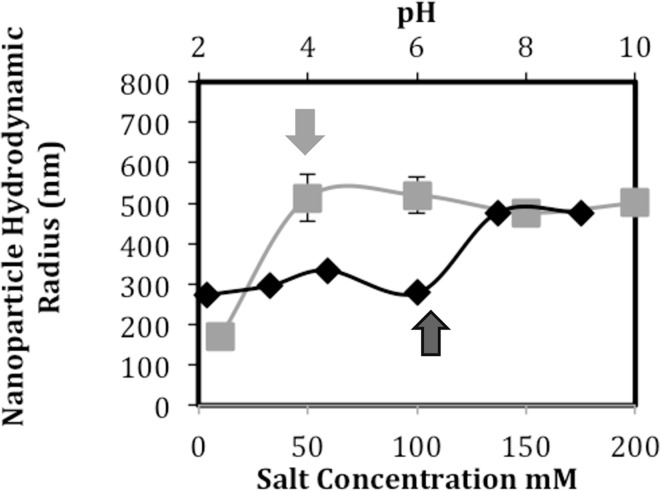
DNA nanoparticle size as a function of the salt content and pH of the PEI solution. PEI concentration was maintained at 4.08 mM in all cases. The pH was ~7.5 at the different salt concentrations, while the salt concentration was 150 mM at the different pH.

Fig 8 shows the hydrodynamic radii of nanoparticles packed in 4.08 mM PEI solutions at different NaCl concentrations but at constant pH (~7.5). Despite the differences in the PEI polyelectrolyte states, the size of the nanoparticles is similar, except for the sample with 10 mM salt concentration. The smaller nanoparticle size in 10 mM NaCl reflects stronger charge-repulsion at low salt-screening conditions that prevents the aggregation of nanoparticles. Overall, our results indicate that in near physiological salt conditions the aggregation state of PEI does not significantly influence the nanoparticle radii.

Fig 8 also shows the hydrodynamic radii of nanoparticles packed in 4.08 mM PEI solutions at different pH at constant (150 mM) salt concentration. These PEI solutions are comparable to those shown in the titration plot of Fig 6. The change of nanoparticle size with pH indicates that polymer charge affects DNA packing. Interestingly, there was no significant change in nanoparticle size between pH = 7.5 and pH = 9. At these two pH values, the PEI polymers have the same charge, but different aggregation levels (Fig 6). The constancy of the nanoparticle size is consistent with our earlier observation namely that DNA-PEI interactions are practically independent of the PEI’s aggregation state. While the size of DNA-PEI particle appears to depend on PEI’s protonation state, more detailed studies are required to understand how other features of PEI polyelectrolyte state (backbone extension, repulsion regime, etc.) affect DNA-PEI interactions. We also note that more rigorous analysis of the DNA arrangement within the nanoparticle is necessary to understand how the PEI state affects nanoparticle packing.

## Summary and Conclusions

PEI is one of the most common polymers used for condensing DNA into nanoparticles for cell transfection and drug delivery applications. It is a cationic polymer with closely spaced charges and an intrinsically hydrophobic backbone. As a result, the polymer is either in aggregated or free form, with different levels of chain extension.

The major conclusions of our study are as follows:

- PEI protonation is relatively independent of its polyelectrolyte state. That is, the protonability of PEI amines is independent of the free vs. aggregated form of PEI.
- The intra-chain repulsion decreases as the salt content increases. The amount of free polymer and its backbone extension are maximized in salt conditions where intra-chain repulsion is high.
- The aggregate form of PEI co-exists with the free polymer and can be separated by filtration. PEI aggregates are the dominant species outside a narrow range of salt concentration. The aggregates exhibit the same charge as the free polymer chains at neutral pH, and act as a buffering agent in the basic pH range by shedding protons.
- PEI buffering occurs by continuous or discontinuous protonation of the amine groups between stalled charge states of 0%, 44% and ~66%.
- The PEI-ninhydrin assay that is commonly used for measuring the amount of amines, actually measures the amount of unprotonated amines.
- The PEI protonation state (but not its aggregation state) significantly affects the size of the condensed DNA nanoparticles and possibly the DNA-PEI interactions.

In the manuscript we report numerical results for the chloride ion only, because it is the most abundant ion in biological systems and it is widely used in packing DNA into nanoparticles for gene and drug delivery. Other studies have reported the effect of different counter-ions on the osmotic and scattering properties of polyelectrolytes like DNA and polyacrylic acid [33,35]. Following the conclusion of these studies, it is reasonable to expect that PEI solutions would exhibit similar behavior in the presence of other monovalent salts unless specific ion adsorption takes place.

The relatively independent protonation and polyelectrolyte properties of PEI can be harnessed for improving its DNA-carrier function and cytotoxicity, and for designing novel biomaterials for environment-sensitive applications.
